# Когнитивные расстройства у пациентов с ожирением и нарушением углеводного обмена (дисгликемией)

**DOI:** 10.14341/probl13389

**Published:** 2024-09-16

**Authors:** Ф. Х. Дзгоева, Е. В. Екушева, В. В. Демидова

**Affiliations:** Национальный медицинский исследовательский центр эндокринологии; Федеральный научно-клинический центр специализированных видов медицинской помощи и медицинских технологий Федерального медико-биологического агентства; Белгородский государственный национальный исследовательский университет; Национальный медицинский исследовательский центр эндокринологии

**Keywords:** ожирение, избыточная масса тела, когнитивные нарушения, сахарный диабет

## Abstract

Ожирение — это хроническое заболевание, гетерогенное по этиологии и клиническим проявлениям, прогрессирующее при естественном течении болезни и характеризующееся избыточным отложением жировой массы в организме. Данное патологическое состояние в последние годы приняло масштаб глобальной эпидемии, размеры которой неуклонно увеличиваются, затрагивая в настоящее время более 2 миллиардов человек во всем мире. Ввиду своей неоднородности, ожирение оказывает негативное влияние на работу практически всех органов и систем организма, способствуя возникновению дополнительных сопутствующих заболеваний и патологических состояний, существенно ухудшающих качество жизни этих пациентов. Так, давно известна тесная взаимосвязь между сахарным диабетом 2 типа и когнитивными нарушениями, как и с рядом других соматических заболеваний: ишемической болезнью сердца, атеросклерозом, неалкогольной жировой болезнью печени, дислипидемией, злокачественными новообразованиями и другими ассоциированными патологическими состояниями на фоне избыточной массы тела и ожирения.

В настоящее время весьма актуальной является проблема возникновения когнитивных нарушений у пациентов с избыточной массой тела или изменением гликемического профиля ввиду высокой распространенности и недостаточной изученности данного вопроса.

## Abstract (en)

Obesity is a chronic disease, heterogeneous in etiology and clinical manifestations, progressing with a natural course, characterized by excessive deposition of fat mass in the body. This pathological condition has taken on the scale of a global epidemic in recent years, which continues to progress steadily, currently affecting more than 2 billion people worldwide. Due to its heterogeneity, obesity has a negative impact on the work of almost all organs and systems of the body, contributing to the emergence of new concomitant diseases and pathological conditions that significantly worsen the quality of life of these patients. Thus, a close relationship between type 2 diabetes mellitus and cognitive impairment has long been known, as well as with a number of other somatic diseases: coronary heart disease, atherosclerosis, non-alcoholic fatty liver disease, dyslipidemia, malignant neoplasms and other associated pathological conditions against the background of overweight and obesity.

Currently, the problem of the relationship of cognitive impairment in patients with overweight or changes in the glycemic profile is very relevant, due to the high prevalence and insufficient study of this issue.

## АКТУАЛЬНОСТЬ

По данным Всемирной организации здравоохранения (ВОЗ), в 2016 г. более 1,9 млрд взрослых людей старше 18 лет имели избыточную массу тела, из которых 650 млн страдали ожирением [1][2]. Кроме того, в ряде исследований было показано, что каждое увеличение индекса массы тела (ИМТ) на 5 единиц выше 25 кг/м² повышает показатели общей смертности на 29%, смертности от сосудистых заболеваний на 41%, а также на 60–120% в связи с диабетом и заболеваниями почек и печени [3]. В настоящее время распространенность пациентов с сахарным диабетом (СД) также достигает неутешительных значений — 366 млн человек, и ожидается, что к 2030 г. это число возрастет до 552 млн [2].

Известно, что само по себе ожирение является коморбидным фактором развития цереброваскулярной патологии, проявляющейся в первую очередь когнитивными нарушениями. В частности, показано, что все составляющие метаболического синдрома (МС) так или иначе ассоциированы с расстройствами когнитивной сферы, в том числе с деменцией [4].

На сегодняшний день в мире почти у 50 млн человек отмечается деменция, и каждый год регистрируется около 10 млн новых случаев этого заболевания [5]. По прогнозам специалистов, к 2030 г. количество людей, страдающих деменцией, удвоится, а к 2050 г. — утроится и будет составлять более 130 млн человек [5]. В большинстве случаев развитию этого патологического состояния на протяжении длительного периода времени предшествуют малозаметные, неспецифические расстройства когнитивных функций, поэтому основное внимание клиницистов должно быть направлено на своевременную диагностику, исключение потенциально курабельных состояний и заболеваний и максимально раннее начало терапии имеющихся недементных нарушений [6].

Когнитивные нарушения (КН) представляют собой снижение одной или нескольких познавательных функций по сравнению с ранее имеющимся уровнем [7]. Они могут иметь разную степень выраженности, наблюдаются при большом количестве заболеваний, и в клинической практике такие пациенты оказываются на приеме у врачей разных специальностей. Различают следующие формы когнитивных нарушений: субъективные КН (СКН); легкие КН (ЛКН), умеренные КН (УКН), деменция (табл. 1) [6][8].

**Table:** Таблица 1. Общая характеристика когнитивных нарушений разной степени выраженности [6][8]

Виды когнитивных нарушений	Характеристика
Субъективные когнитивные нарушения	Жалобы на стойкое ухудшение умственной работоспособности по сравнению с ранее имеющейся, возникшее без видимой причин; отсутствие отклонений от возрастной нормы при выполнении стандартных нейропсихологических тестов для диагностики когнитивных нарушений; они не вызывают профессиональную, социальную или бытовую дезадаптацию
Легкие когнитивные нарушения	Наличие жалоб и снижение одной или нескольких познавательных функций, о которых говорит пациент и которые может выявить специалист с помощью специальных нейропсихологических тестов; они не вызывают профессиональную, социальную или бытовую дезадаптацию
Умеренные когнитивные нарушения	Наличие жалоб и снижение одной или нескольких познавательных функций, о которых сообщает пациент и/или окружающие люди и которые объективно выявляются с помощью нейропсихологических тестов. Изменения не вызывают профессиональной, социальной или бытовой дезадаптации, но заметно влияют на качество жизни пациента. Нет признаков деменции
Деменция	Выраженное и устойчивое нарушение одной или нескольких познавательных функций, которое приводит к профессиональной, бытовой и социальной дезадаптации пациента и подтверждается с помощью нейропсихологических тестов; отмечается на фоне ясного сознания

Поскольку в настоящее время проведены исследования с небольшим числом больных, то сложно иметь объективную картину представленности когнитивных расстройств у пациентов с ожирением или СД2. Вместе с тем имеющиеся данные позволяют предположить существенную распространенность таких патологических состояний у этих людей.

## КОГНИТИВНЫЕ РАССТРОЙСТВА У ПАЦИЕНТОВ С ОЖИРЕНИЕМ И НАРУШЕНИЕМ ГЛИКЕМИЧЕСКОГО ПРОФИЛЯ

Ожирение является часто наблюдаемой и актуальной медико-социальной проблемой во всем мире и тесно связано с рядом сопутствующих заболеваний и патологических состояний, в частности с развитием КН, что неоднократно продемонстрировано в ряде исследований не только у пациентов с ожирением, но и с избыточной массой тела (табл. 2) [9][10][11][12].

**Table:** Таблица 2. Исследования, изучающие когнитивные нарушения у лиц с ожирением и нарушением гликемического профиля Примечание. 1) ИТБ — индекс соотношения объема талии/бедер, 2) ИМТ — индекс массы тела.

Авторы исследования	Тип клинического исследования/количество исследований и участников	Основные выводы
Hartanto A., Yong J.C., et al. [9]	Лонгитудинальное исследование (n=2652)	Обнаружена прямая корреляция между снижением эпизодической памяти и наличием ожирения, что более значимо демонстрирует важность анализа показателя ИТБ¹, а не ИМТ²
Pedditzi E., Peters R., et al. [10]	Систематический обзор и метаанализ (21 исследование, n=62 425)	Показана положительная связь между ожирением и развитием деменции
Anstey K.J., Cherbuin N., et al. [11]	Систематический обзор и метаанализ (16 исследований, из которых 15 — проспективные)	Выявлена положительная корреляция между недостатком или избытком массы тела или ожирением и нарушением когнитивных функций
Tuligenga R.H., Dugravot A., et al. [14]	Ретроспективный анализ когортного лонгитудинального исследования (n=5653)	Выявлена прямая зависимость между наличием сахарного диабета и более быстрым снижением когнитивных функций (способности логично и последовательно рассуждать, ухудшения объема памяти, общим нарушением познавательной сферы)
Palta P., Carlson M.C., et al. [15]	Рандомизированное двойное слепое плацебо-контролируемое исследование (n=3069)	Показана прямая корреляция между сахарным диабетом и низкими показателями при тестировании исполнительных функций и фонематической вербальной беглости
Rawlings A.M., Sharrett A.R., et al. [16]	Проспективное обсервационное когортное исследование (n=15 792)	Продемонстрирована прямая зависимость между сахарным диабетом и развитием когнитивных расстройств
Kinattingal N., Mehdi S., et al. [17]	Обсервационное кросс-секционное исследование (n=200)	Показана прямая корреляционная связь между наличием сахарного диабета 2 типа и развитием КН
Старостина Е.Г., Володина М.Н. и др. [18]	Пилотное проспективное открытое наблюдательное исследование (n=178)	Продемонстрирована прямая зависимость между наличием депрессии, когнитивных нарушений и 5-летней смертностью у пациентов с СД2
Raji C.A., Ho A.J., et al. [19]	Нерандомизированное контролируемое исследование (n=94)	Продемонстрирована прямая связь между избыточной массой тела, гиперинсулинемией и развитием СД2 и возникновением церебральной атрофии в лобных, височных и подкорковых областях головного мозга
Gannon O.J., Robison L.S., et al. [22]	Доклиническое исследование на мышах (n=251)	Обнаружена прямая связь диеты с высоким содержанием жиров и развитием преддиабета, избыточной массой тела и КН
Smith P.J., Mabe S., et al. [29]	Рандомизированное клиническое исследование (n=160)	Продемонстрирована обратная связь между избыточной массы тела и ожирением и результатами когнитивного тестирования
Feinkohl I., Janke J., et al. [30]	Перекрестное исследование (n=669)	Выявлена обратная корреляция между уровнем лептина, соотношением лептин/адипонектин и степенью выраженности КН

Hartanto A., Yong J.C. [9] продемонстрировали достоверную обратную связь между ожирением и объемом кратковременной памяти, вместе с тем при снижении массы тела исследуемых наблюдалось улучшение исполнительных функций. Также полученные в исследовании данные [9] позволяют предположить важную роль церебральных структур в регуляции пищевого поведения, возникновении переедания и сложности контроля веса тела, что, безусловно, увеличивает риск развития ожирения в дальнейшем. Следует отметить, что пациенты на момент анализа также являлись участниками Когнитивного проекта в рамках второго (II) и третьего (III) этапов MIDUS (англ. Midlife Development in the United States — Рост среднего возраста в США). Кроме того, отмечалась достоверная положительная корреляция между бóльшим показателем индекса соотношения объема талии/бедер (ИТБ) и выраженностью КН в отличие от ИМТ. Эти результаты, возможно, позволят переоценить значение показателя ИМТ как основного при анализе осложнений у пациентов с избыточной массой тела и ожирением [9].

Другой метаанализ [10] обнаружил высокую положительную корреляцию между ожирением и неврологическими расстройствами, такими как болезнь Альцгеймера (БА) и другие виды деменции (95% доверительный интервал (ДИ): 1,20–1,66). Вместе с тем данный показатель не имел статистической значимости у лиц пожилого возраста, что, возможно, связано с разными инструментами для оценки КН, а также меньшим количеством лиц с ожирением и повышенным ИМТ, что часто обусловлено развитием старческой астении в данной возрастной группе [10].

Взаимосвязь между показателем ИМТ у лиц среднего и пожилого возраста и развитием деменции была исследована в метаанализе Anstey K.J., Cherbuin N. [11], где была продемонстрирована положительная корреляция между недостатком, избытком массы тела и ожирением с развитием КН, в то время как стабильная масса тела в пожилом возрасте обнаружила противоположную взаимосвязь [11]. Помимо вышеуказанного, исследуемые с избыточной массой тела имели на 33% больший риск возникновения СД2 в пожилом возрасте. Важно отметить, что наличие ожирения в среднем возрасте было связано с увеличением риска развития БА в 3,08 раза для женщин и в 2,45 раза для мужчин соответственно.

Yang Y., Shields G.S. в своем метаанализе обнаружили существенное нарушение исполнительных функций у обследуемых с избыточной массой тела и ожирением по сравнению с группой контроля [12], причем у участников с избыточной массой тела наблюдалось снижение объема рабочей памяти, когнитивной гибкости, нарушение способности к планированию и принятию решений, как и беглости речи.

В поперечном исследовании с участием 224 пожилых женщин (средний возраст 65,69± 3,7 лет) [13] оценивалась взаимосвязь между наличием избыточной массы тела или ожирения и изменениями исполнительных функций в трех группах: с нормальной, избыточной массой и ожирением. Согласно результатам теста прокладывания пути (TMT), выявлена отрицательная корреляционная связь между ИМТ и адекватным исполнительным функционированием в пожилом возрасте [13].

В когортном исследовании Whitehall I.I. [14] у пациентов с СД по сравнению с участниками с нормогликемией было продемонстрировано более быстрое ухудшение возможности запоминания и воспроизведения — на 45% (10-летняя разница в снижении -0,13 SD, 95% ДИ от -0,26 до -0,00; p=0,046), способности логично и последовательно рассуждать — на 29% (-0,10 SD, от -0,19 до -0,01; p=0,026) и в целом более низкие баллы при оценке когнитивных функций — на 24% (-0,11 SD, от -0,21 до -0,02, p=0,014). Плохой гликемический контроль у пациентов с установленным диабетом был также сопряжен со значительно более быстрым ухудшением КН [14].

В исследовании GEMS (англ. The Ginkgo Evaluation of Memory Study — Оценка влияния Гинкго на память) [15] продемонстрирована прямая корреляция между СД и низкими показателями при тестировании исполнительных функций и фонематической вербальной беглости, при этом не было выявлено значимого снижения других когнитивных функций по сравнению с пациентами без СД, что противоречит данным ряда предыдущих исследований, например, ARIC (англ. Atherosclerosis Risk in Communities — Риск атеросклероза в сообществах) [16], что может быть связано с разными возрастными категориями участников (57 лет в ARIC и 78 лет в GEMS).

Анализ КН у пациентов с СД с помощью шкалы MoCA (англ. Montreal Cognitive Assessment — Монреальская шкала для оценки когнитивных функций) [17] продемонстрировал значительную распространенность нарушений по сравнению с группой контроля: 18,99±0,48 и 26,21±0,46 баллов соответственно.

Изучение взаимосвязи аффективных и когнитивных нарушений у пациентов с СД2 и показателя 5-летней смертности обнаружило прямую корреляцию между выраженными КН и 25% прогнозируемой вероятностью смертельного исхода; как и выраженными депрессивными расстройствами и аналогичного показателя — 26% [18]. Напротив, нормальное когнитивное функционирование и отсутствие аффективных нарушений минимально влияет на анализируемый показатель: 2% и 2% соответственно, что подчеркивает необходимость выявления и лечения данных расстройств у пациентов с СД2.

## ПАТОФИЗИОЛОГИЧЕСКИЕ ИЗМЕНЕНИЯ ГОЛОВНОГО МОЗГА У ПАЦИЕНТОВ С ОЖИРЕНИЕМ И НАРУШЕНИЕМ ГЛИКЕМИЧЕСКОГО ПРОФИЛЯ

Жировая ткань, являясь гормонально активной, продуцирует ряд провоспалительных факторов, которые приводят к развитию разнообразных и многоуровневых патофизиологических изменений, в том числе к развитию системного воспаления. Так, в работе Raji C.A., Ho A.J. [19] использование магнитно-резонансной морфометрии для оценки состояния серого и белого вещества головного мозга у пожилых пациентов с нормальными когнитивными функциями позволило продемонстрировать взаимосвязь между избыточной массой тела, гиперинсулинемией, развитием СД2 и наличием атрофии в лобных, височных и подкорковых церебральных областях. В этом же исследовании [19] у пациентов с ожирением выявлены атрофические изменения в области передней поясной извилины, гиппокампе и таламусе (рис. 1). Следует отметить, что у пациентов с избыточной массой тела и без выраженных КН наблюдались схожие патологические изменения в головном мозге [19].

**Figure:**
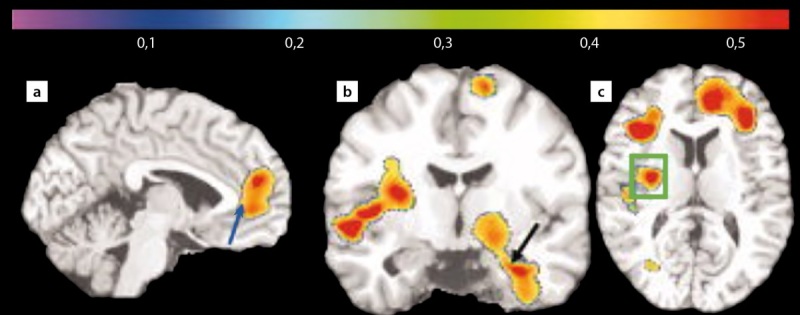
Рисунок 1. Уменьшение объема серого и белого вещества в лобных отделах, передней поясной извилине (а — синяя стрелка), в гиппокампе (b — черная стрелка) и базальных ганглиях (c — зеленая рамка) у пациентов с ожирением по данным магнитно-резонансной морфометрии [19].

В обзоре Schmitt L.O., Gaspar J.M. [20] показано, что при ожирении наблюдается митохондриальная дисфункция, сопряженная с повышенным уровнем активных форм кислорода (АФК) и оксида азота (NO), снижением содержания белка — коактиватора 1-альфа-рецептора (PGC-1α), митофузинов 1 и 2 (Mfn1/Mfn2) и мембранного потенциала митохондрий (ΔΨm) (рис. 2). Все эти патологические изменения ассоциированы с развитием системного воспаления, сопровождающегося повышенной экспрессией ряда провоспалительных цитокинов, в частности интерлейкина-6 (ИЛ-6), интерлейкина-1 бета (ИЛ-1β) и некроза опухоли-α (ФНО-α) [20], и нарушением проницаемости гематоэнцефалического барьера (ГЭБ), что в свою очередь приводит к проникновению провоспалительных цитокинов в паренхиму головного мозга и дальнейшей активации глиальных клеток (микроглии и астроцитов) [21]. Активированная микроглия секретирует большое количество воспалительных цитокинов (TNFα, IL-1β и IL-6), поддерживая системное воспаление и приводя к еще большему повреждению нейронов [21].

**Figure:**
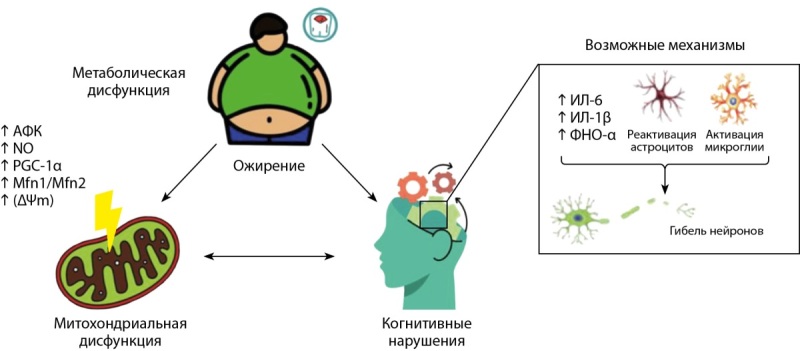
Рисунок 2. Роль ожирения в развитии системного воспаления и митохондриальной дисфункции [20]. Примечание: АФК — активные формы кислорода; NO — оксид азота; ΔΨm — мембранный потенциал митохондрий; PGC-1α — белок — коактиватор 1-альфа-рецептора; Mfn1/Mfn2 — митофузины 1 и 2; ИЛ-6 — интерлейкин-6; ИЛ-1β — интерлейкин 1-бета; ФНО-α — фактор некроза опухоли-альфа.

Гиппокамп — это функционально значимое образование головного мозга, часть лимбической системы, в первую очередь связанная с механизмами обучения, внимания, различных модальностей памяти и регуляцией эмоциональных реакций [19]. Нарушения функционирования гиппокампа наблюдаются на ранних стадиях нейродегенеративного процесса, включая сосудистую деменцию и болезнь Альцгеймера [19].

Gannon O.J., Robison L.S. продемонстрировали наличие прямой корреляции между уровнем системного воспаления в гиппокампе, вызванного ожирением на фоне высококалорийного питания, и нарушениями обучения и памяти [22]. В вышеуказанном исследовании мышей разделили на группы и кормили пищей с высоким (60%) или низким содержанием (10%) жира. У обоих полов из первой группы обнаружено развитие предиабетического фенотипа (нарушение толерантности к глюкозе) и увеличение массы тела. Примечательно, что у самок, помимо наличия более широкого спектра КН (нарушение эпизодической и пространственной памяти, пространственного обучения), были обнаружены выраженные проявления астроглиоза и отложение белка Aβ (англ. amyloid beta — бета-амилоид) по сравнению с самцами [22].

## РОЛЬ ИНСУЛИНА В РАЗВИТИИ КОГНИТИВНЫХ НАРУШЕНИЙ

Инсулин является важным регулятором гомеостаза и метаболизма глюкозы. Однако его роль в функционировании центральной нервной системы и в частности возникновении КН в настоящий момент является полностью не изученной (рис. 3) [23]. Так, в своей работе Sims-Robinson C., Kim B. высказали предположение о том, что при проникновении в головной мозг через гематоэнцефалический барьер инсулин подвергается воздействию специфического фермента IDE (aнгл. Insulin-Degrading Enzyme — фермент, разрушающий инсулин), который также способен расщеплять белок Aβ, играющий ключевую роль в развитии болезни Альцгеймера [24]. Таким образом, инсулин и белок Aβ конкурируют за связывание с IDE, поэтому в состоянии гиперинсулинемии происходит связывание инсулина с IDE и накопление белка Aβ, что, возможно, в дальнейшем является одним из факторов риска развития болезни Альцгеймера (рис. 4) [23–25].

**Figure:**
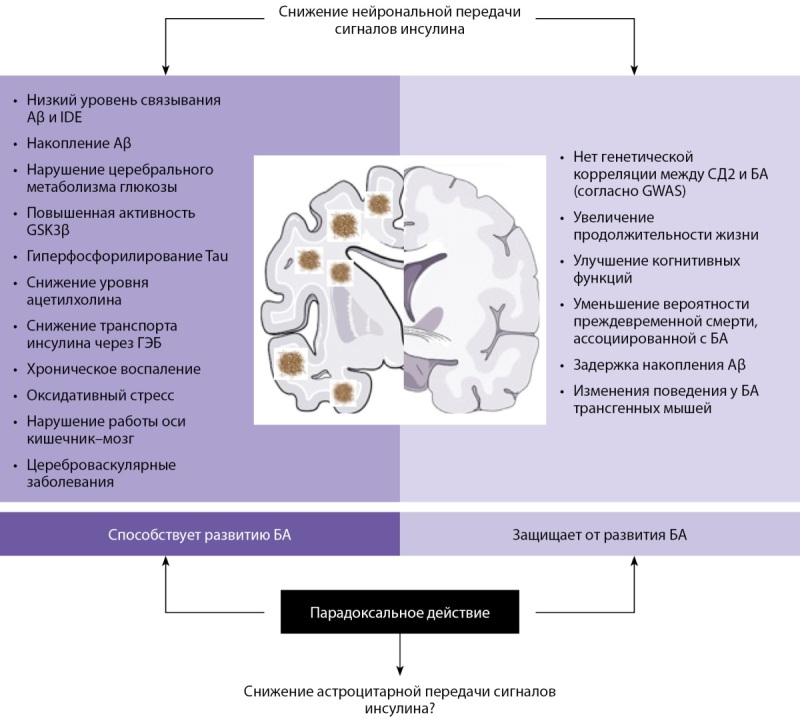
Рисунок 3. Основные механизмы влияния инсулина на патогенез болезни Альцгеймера [23]. Примечание: Aβ — амилоид-бета; IDE — фермент, разрушающий инсулин; GSK-3β — киназа гликогенсинтазы-3-бета; Tau — белок Tau; ГЭБ — гематоэнцефалический барьер; СД2 — сахарный диабет 2 типа; БА — болезнь Альцгеймера; GWAS — исследование полногеномного поиска ассоциаций.

**Figure:**
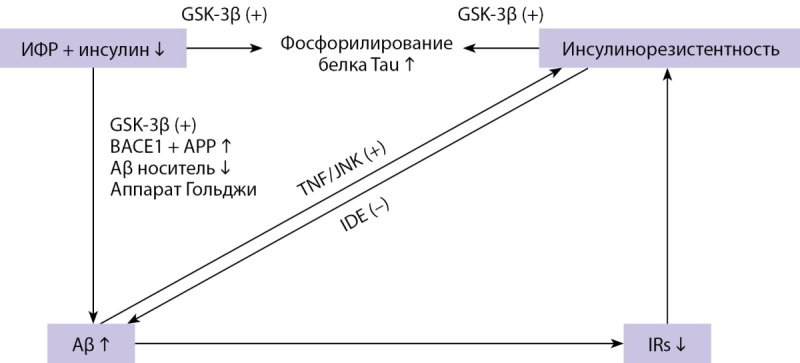
Рисунок 4. Роль инсулинорезистентности и дефицита инсулина в развитии болезни Альцгеймера [25]. Примечание: BACE1 — бета-секретаза-1; GSK-3β — киназа гликогенсинтазы-3-бета; IDE — фермент, разрушающий инсулин; IRs — субстраты рецептора инсулина; TNF — фактор некроза опухоли; JNK — N-концевая киназа c-Jun; APP — белок —предшественник амилоида; Aβ — амилоид-бета; ИФР — фактор роста инсулина.

Помимо вышеуказанного, гиперинсулинемия может способствовать прогрессированию системного воспаления и окислительного стресса за счет ингибирования АМФ-активируемой протеинкиназы [23]. Примечательно, что высокие уровни провоспалительных цитокинов, таких как интерлейкин-1β, интерлейкин-6 и гамма-интерферон, расположены вблизи бляшек Aβ и клеток-макрофагов, что указывает на важную роль системного воспаления в патологии болезни Альцгеймера [23].

Большой интерес в последнее время представляет собой влияние инсулина на астроциты, ввиду того что являются наиболее распространенными клетками головного мозга и имеют тесную связь с нейронами [26]. Они поддерживают оптимальную микросреду, обеспечивающую адекватное функционирование нейронов, участвуют в нейрогенезе, регуляции тонуса сосудов головного мозга, поддержании ГЭБ и регуляции энергетического гомеостаза [26]. Помимо вышеуказанного, астроциты под воздействием инсулина могут реагировать на изменения уровня нейротрансмиттеров, вызывая повышение внутриклеточного кальция, а также способны выделять ряд глиотрансмиттеров, обеспечивающих взаимодействие между нейронами и глиальными клетками [23]. В 2018 г. доклиническое исследование Cai W., Xue C. впервые продемонстрировало развитие у мышей с выключенными рецепторами инсулина поведенческих реакций в виде тревожного поведения и депрессивного состояния. Вышеуказанные изменения оценивались с помощью ряда валидизированных тестов, отражающих настроение и возникновение депрессии или тревожности у грызунов (тест с сахарозой, принудительного плавания, новизны) [27]. Авторы предположили, что неспособность восприятия рецепторами сигналов инсулина нарушило в астроцитах мышей фосфорилирование тирозина Munc18c и, по-видимому, снизило экзоцитоз АТФ из астроцитов. Данные изменения привели к уменьшению высвобождения дофамина из прилегающего к ним ядра, что оказало прямое воздействие на функциональное состояние нейронных связей, участвующих в процессе обучения и в формировании эмоционально-поведенческих реакций [27].

Несмотря на данные, свидетельствующие о важной роли инсулинорезистентности в возникновении когнитивных нарушений, ряд работ продемонстрировали возможности положительного влияния дефицита инсулина на развитие вышеуказанных патологических изменений [23]. Так, в доклинических исследованиях было продемонстрировано, что угнетение рецепторов инсулина и невозможность в этой связи передачи его сигналов, может способствовать замедлению процессов старения [23]. Выключение рецепторного инсулинового аппарата также было ассоциировано со снижением рисков преждевременной смерти, связанной с болезнью Альцгеймера и, что немаловажно отметить, с более медленным накоплением белка Aβ [23].

## ВЛИЯНИЕ АДИПОКИНОВ НА РАЗВИТИЕ КОГНИТИВНЫХ НАРУШЕНИЙ

Среди адипокинов, секретируемых жировой тканью и оказывающих влияние на развитие КН, в большей степени изучены лептин и адипонектин. Оба вышеуказанных гормона жировой ткани способны проникать через ГЭБ и участвовать в процессе системного воспаления [29].

Smith, Patrick J. проанализировали участников (n=160; возраст ≥55 лет с избыточной массой тела или ожирением, без КН) исследования ENLIGHTEN (англ. Exercise and NutritionaL Interventions for coGnitive and Cardiovascular HealTh ENhancement — упражнения и диетические рекомендации для улучшения когнитивного и сердечно-сосудистого здоровья) [29]. Когнитивные способности оценивались с использованием 45-минутного тестирования с использованием тестов, оценивающих исполнительную функцию, вербальную и зрительную память. Согласно полученным результатам, более высокие уровни ИМТ были связаны с худшими показателями исполнительных функций (β=-0,16, p=0,024), вербальной памяти (β=-0,16, p=0,030) и более высокими уровнями индекса HOMA-IR (англ. Homeostasis model assessment of insulin resistance — Модель гомеостаза для оценки резистентности к инсулину) и лептина в сыворотке крови, в частности у женщин (p<0,001). [29]. Авторы предполагают, что вышеуказанные КН связаны с персистирующим повышением уровня лептина в крови у пациентов с ожирением. Данные изменения способствуют нарушению передачи внутриклеточных сигналов от лептинового рецептора, что в дальнейшем может приводить к развитию лептинорезистентности и снижению когнитивных функций [29].

В перекрестном исследовании Feinkohl I., Janke J. c участием 669 пациентов европеоидной расы в возрасте ≥65 лет BioCog (англ. Biomarker Development for Postoperative Cognitive Impairment in the Elderly — Разработка биомаркеров послеоперационных когнитивных нарушений у пожилых людей) была выявлена обратная корреляция между уровнем лептина (1,33; 95% ДИ 1,05, 1,69; р=0,02), соотношением лептин/адипонектин (1,26; 95% ДИ 1,01, 1,57; р=0,04) и степенью выраженности КН [30]. Работа проводилась среди пациентов, перенесших оперативное вмешательство (длительность операции ≥60 минут), у которых когнитивные функции оценивались в шести нейропсихологических тестах. Вместе с тем ИМТ не коррелировал со степенью выраженности данных изменений [30].

## ЗАКЛЮЧЕНИЕ

Таким образом, анализ многочисленных исследований последних лет убедительно показал несомненную важность изучения когнитивной сферы у пациентов с ожирением и нарушением гликемического профиля. Несмотря на высокий уровень доказательности и правильный дизайн проведенных исследований, в дальнейшем необходимо обращать внимание на такие факторы, как пол, расовая/этническая принадлежность, тяжесть СД, состояние когнитивных функций при впервые выявленном диабете, длительно существующем заболевании и при его дебюте в раннем возрасте, а также более тщательно оценивать у пациентов отдельные когнитивные функции и другие патологические изменения на фоне СД. Важный акцент необходимо сделать на выявлении специфических ранних предикторов развития когнитивных нарушений у пациентов с ожирением и дисгликемией для создания специализированных методов профилактики их дальнейшего прогрессирования.

## ДОПОЛНИТЕЛЬНАЯ ИНФОРМАЦИЯ

Источники финансирования. Работа выполнена по инициативе авторов без привлечения финансирования.

Конфликт интересов. Авторы декларируют отсутствие явных и потенциальных конфликтов интересов, связанных с содержанием настоящей статьи.

Участие авторов. Дзгоева Ф.Х. — концепция и дизайн статьи, переработка первого варианта статьи на предмет важного интеллектуального содержания, окончательное утверждение рукописи; Екушева Е.В. — переработка нескольких вариантов статьи на предмет важного интеллектуального содержания, редактирование текста статьи; Демидова В.В. — анализ данных и литературы, редактирование текста статьи. Все авторы одобрили финальную версию статьи перед публикацией, выразили согласие нести ответственность за все аспекты работы, подразумевающую надлежащее изучение и решение вопросов, связанных с точностью или добросовестностью любой части работы.
